# Supplementary material for: Investigation of Newly Synthesized Bis-Acyl-Thiourea Derivatives of 4-Nitrobenzene-1,2-Diamine for Their DNA Binding, Urease Inhibition, and Anti-Brain-Tumor Activities
Source: Molecules. 2023 Mar 16;28(6):2707. doi: 10.3390/molecules28062707 (PMC10051851; doi:10.3390/molecules28062707)
Supplement: Supplementary file 1 [file molecules-28-02707-s001.zip › molecules-2248173-supplementary.pdf]

Supplementary data

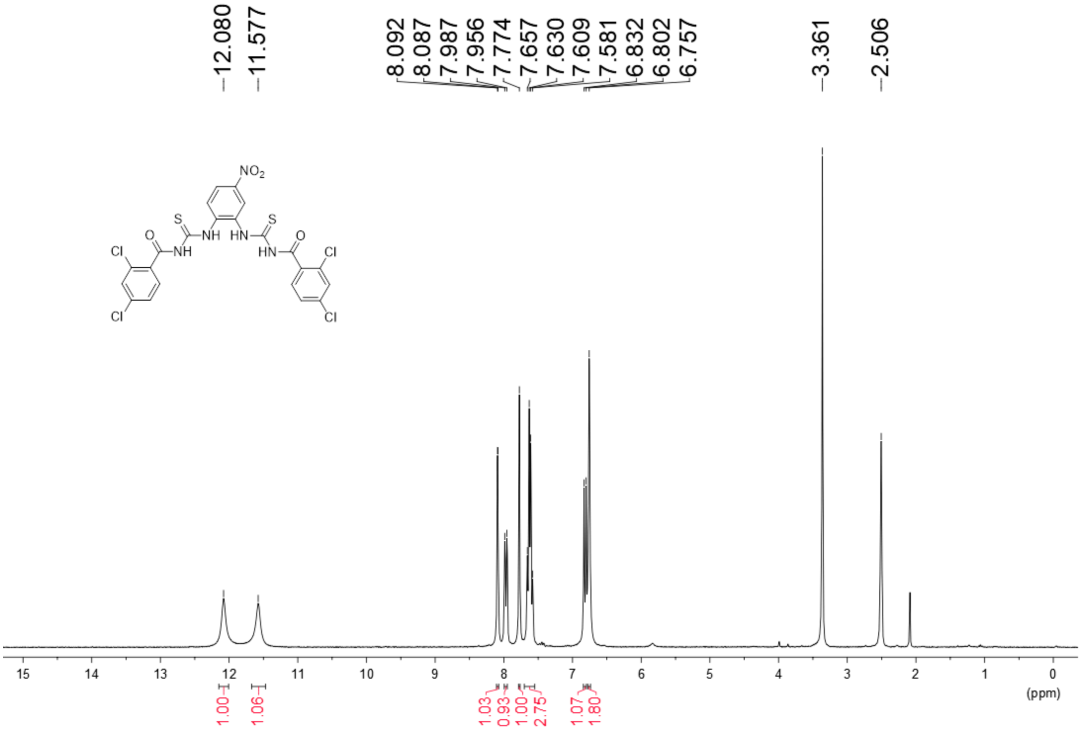

Figure S1. ^{1}H-NMR of UP-1.

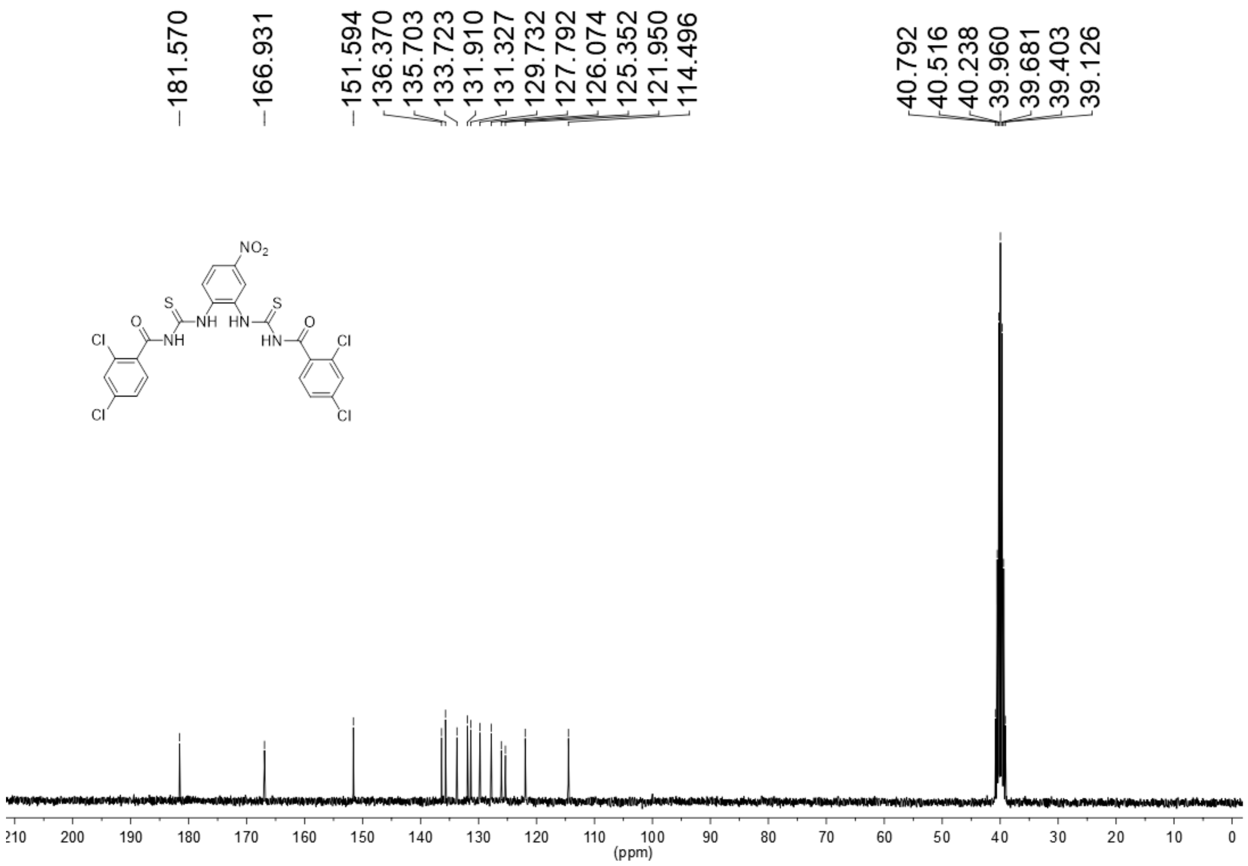

Figure S2. ^{13}C-NMR of UP-1.

FT-IR of UP-1

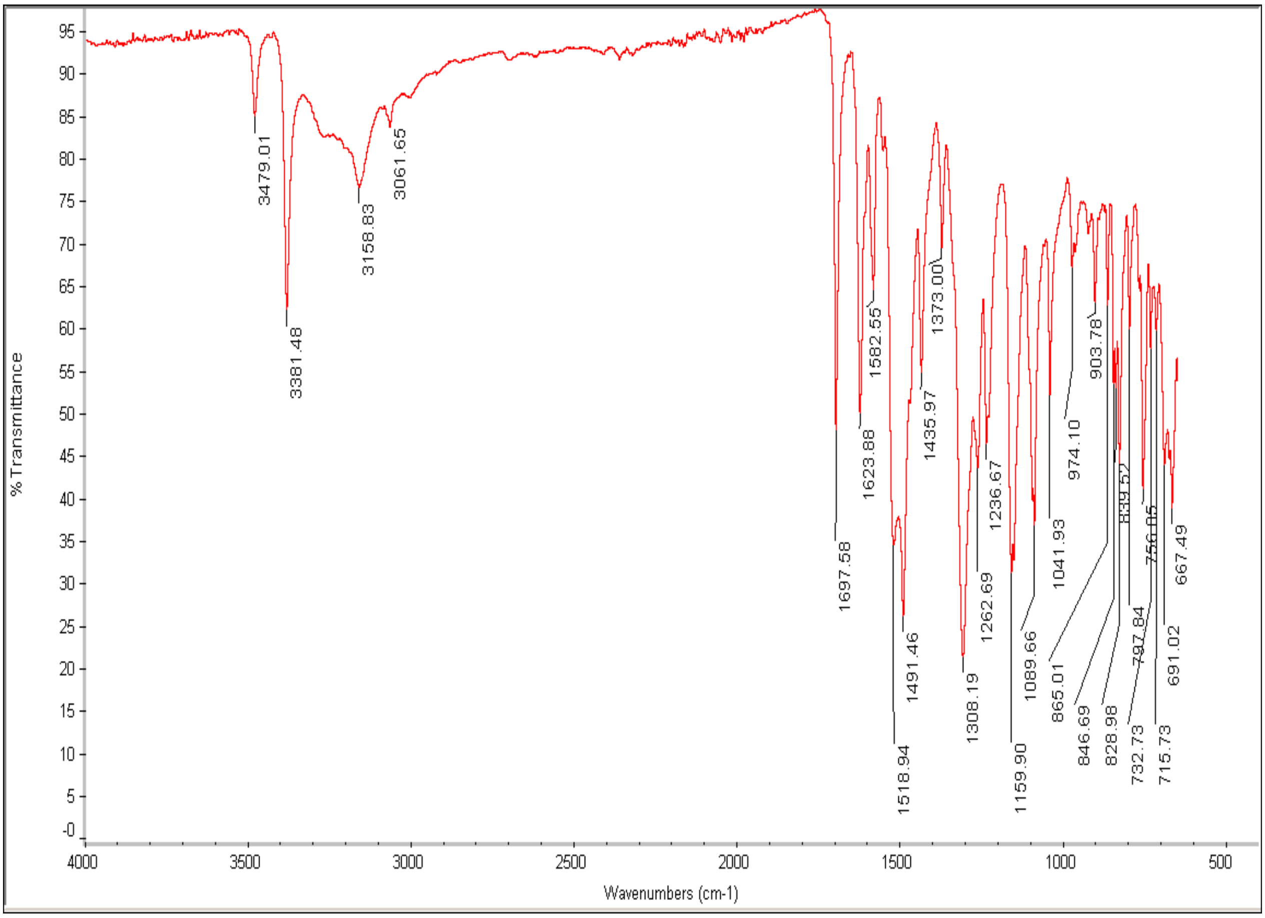

Figure S3. FT-IR of UP-1.

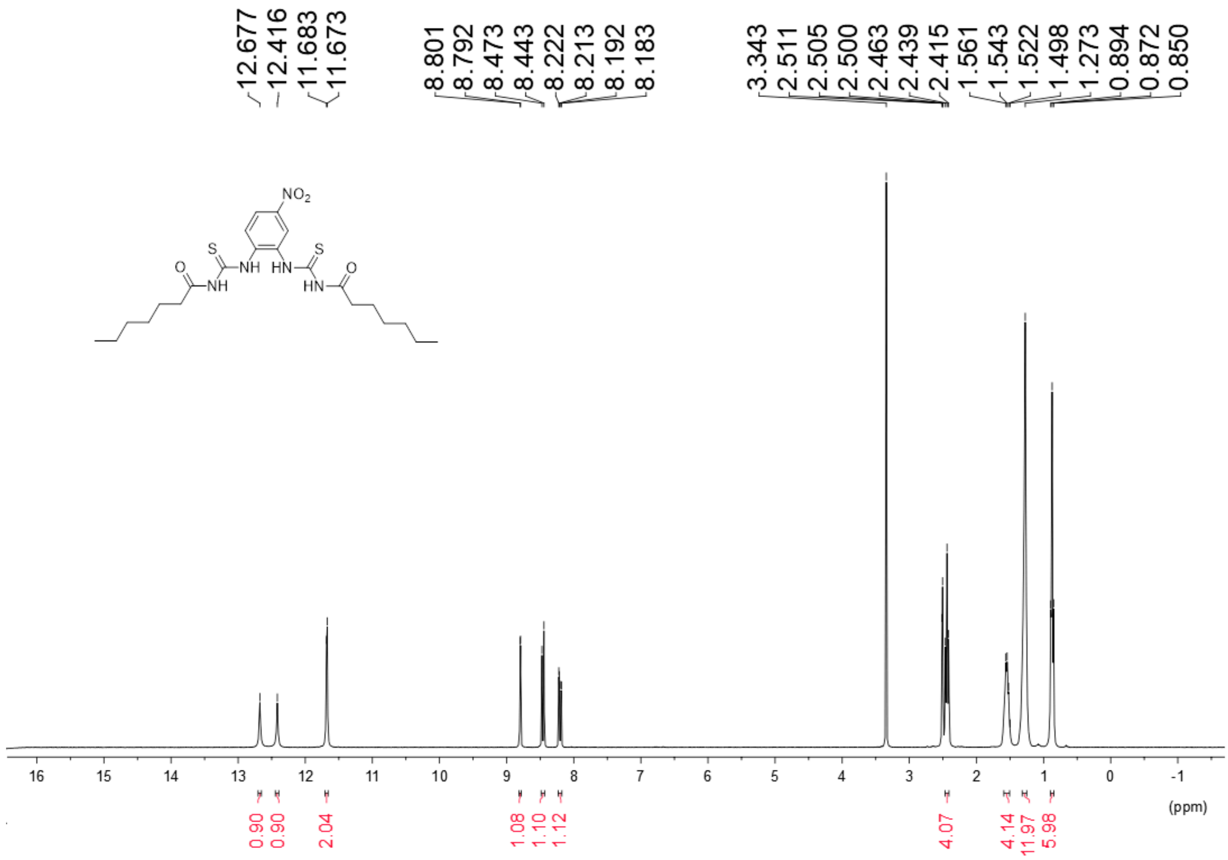

Figure S4. H^1 NMR for UP-2.

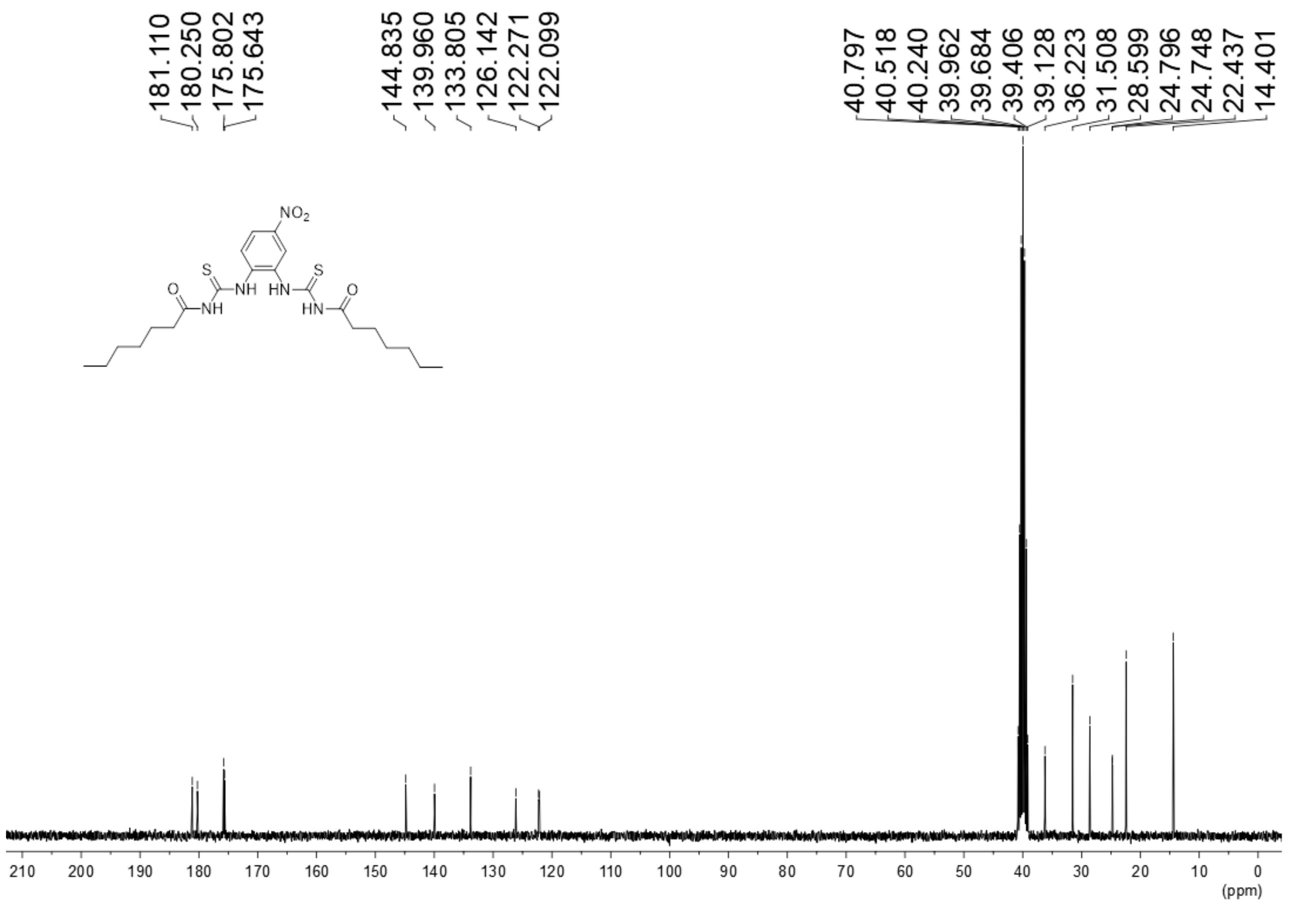

Figure S5. C^{13} NMR for UP-2.

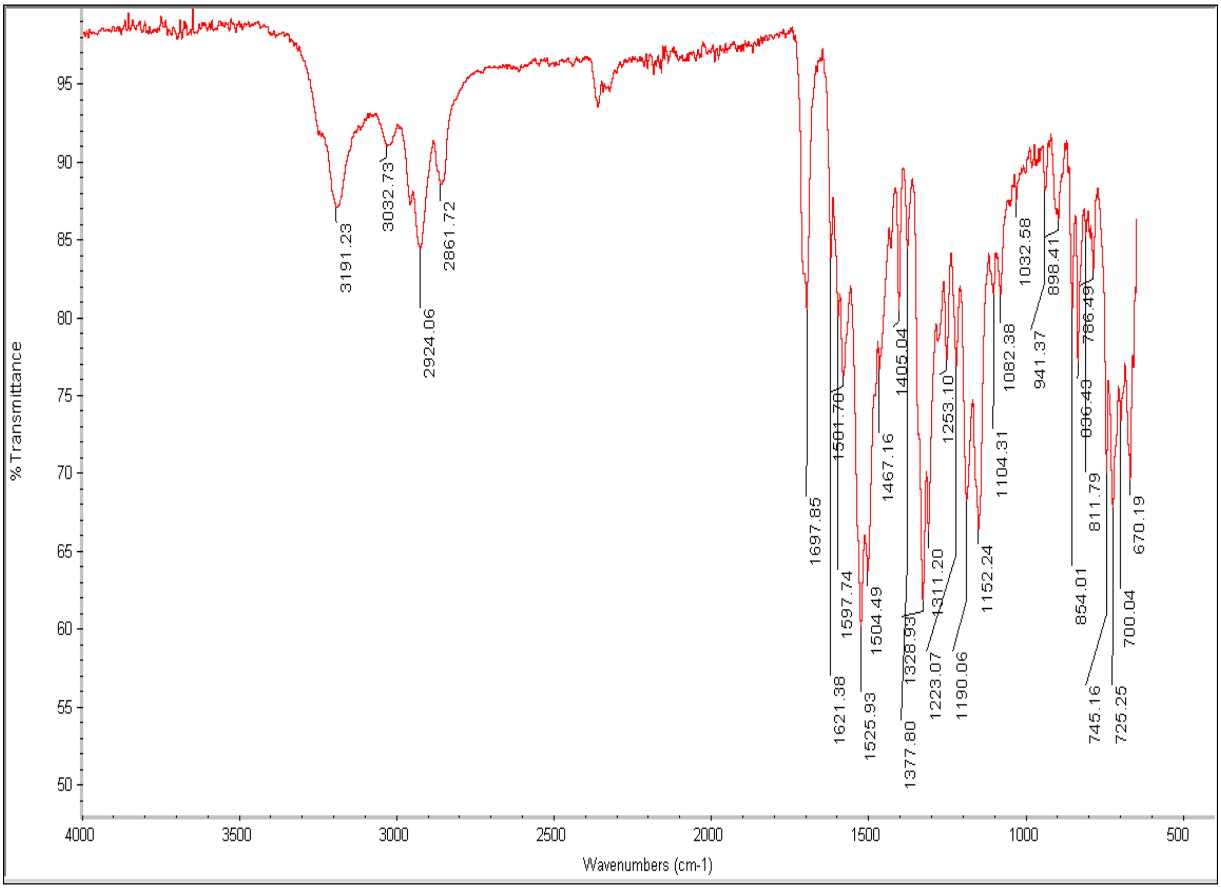

Figure S6. FT-IR of UP-2.

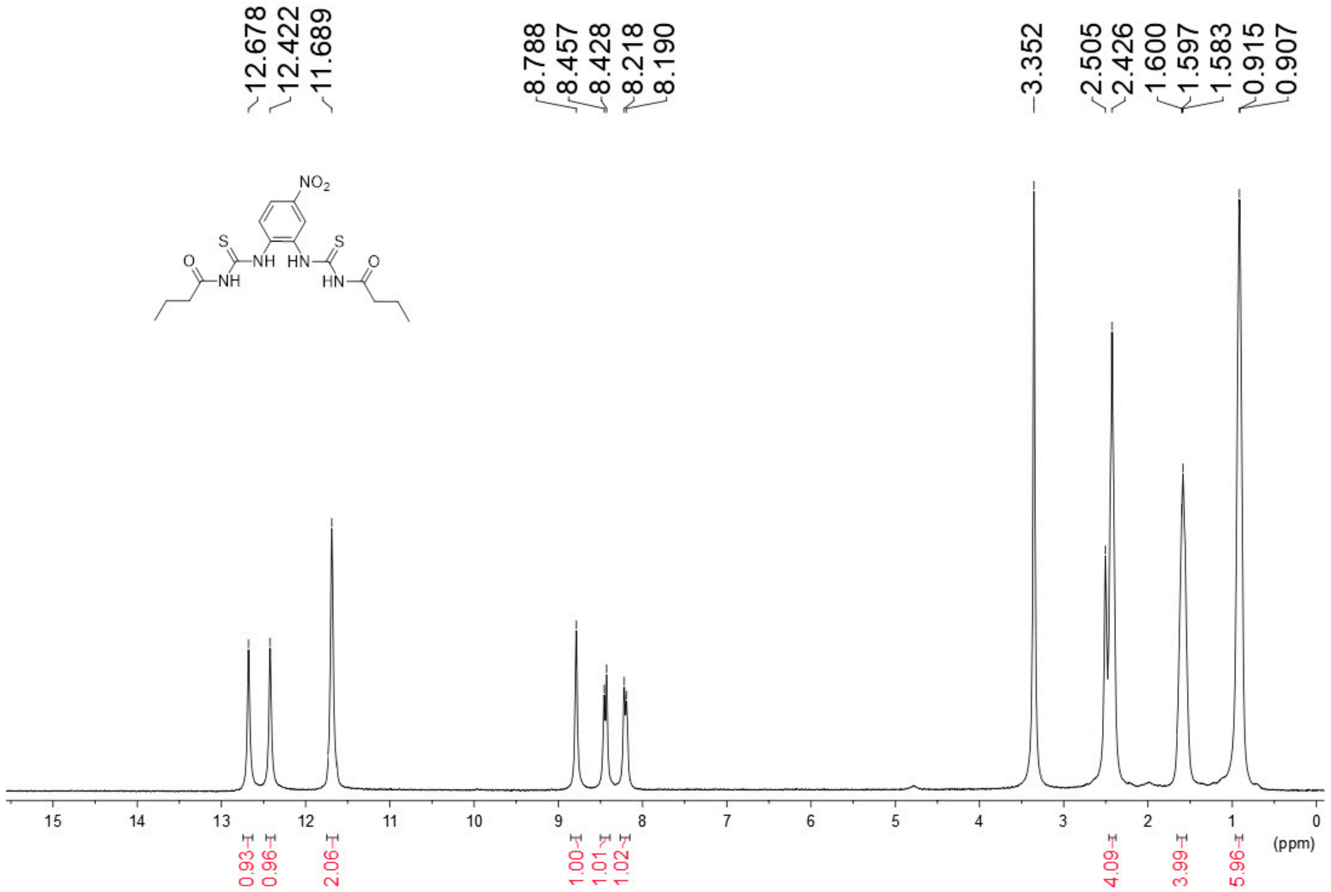

Figure S7. H^1 NMR for UP-3.

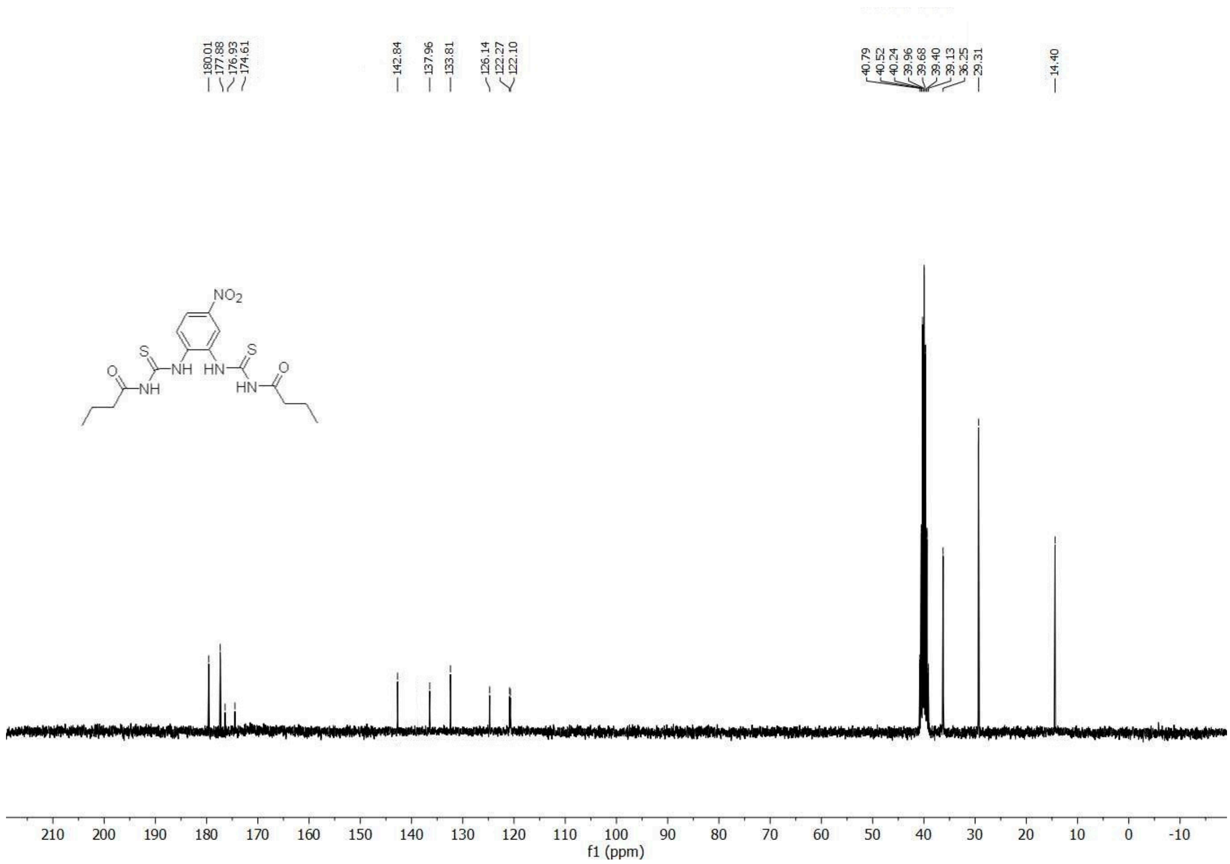

Figure S8. C^{13} NMR for UP-3.

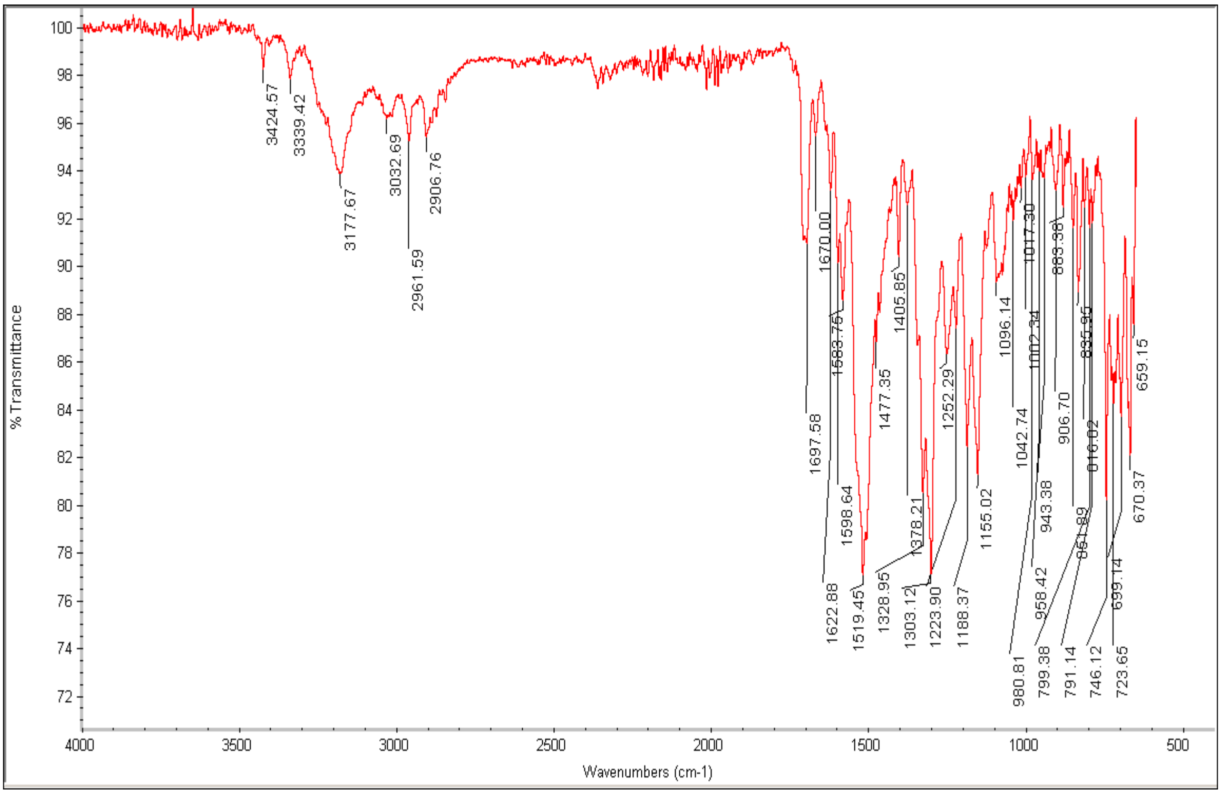

Figure S9. FTIR for UP-3.

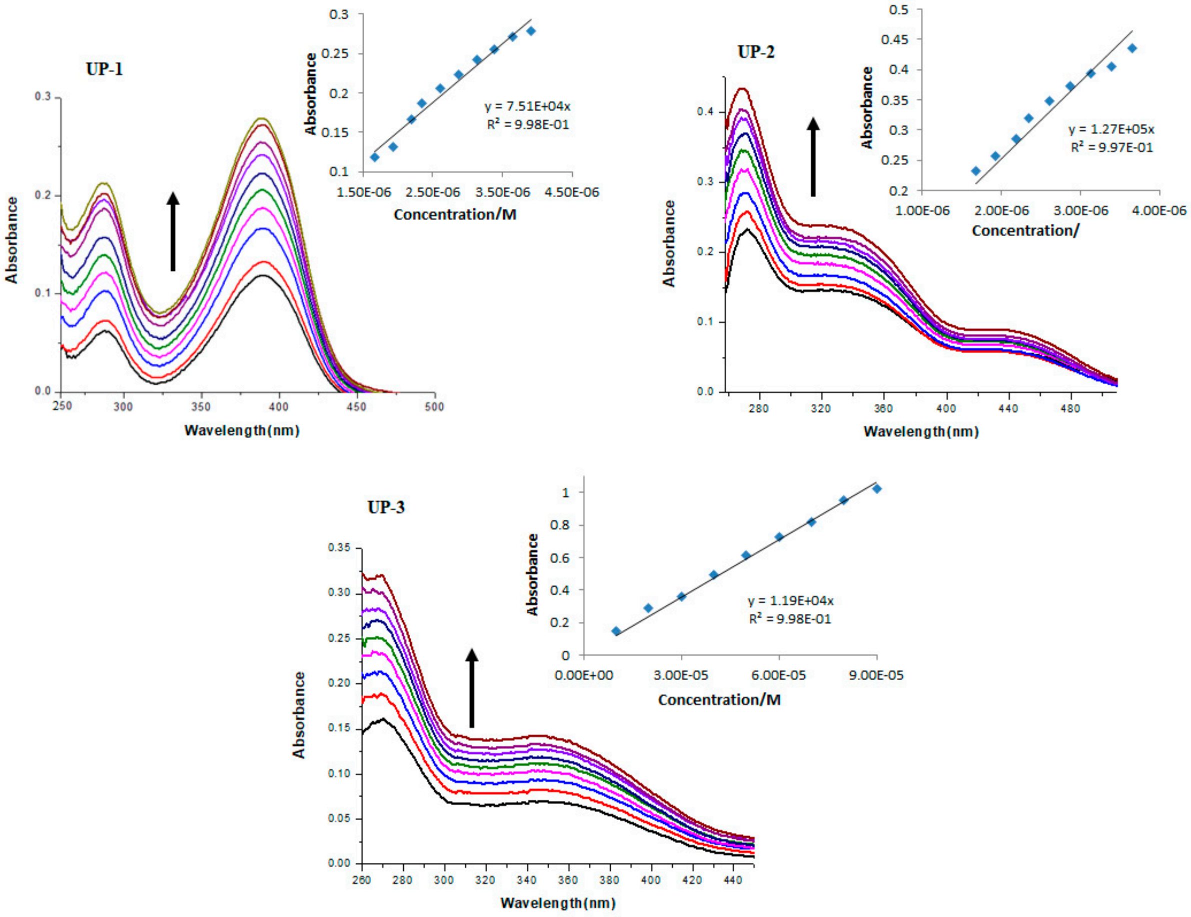

Figure S10. Concentration profile of the compounds, inset: Absorbance vs. Concentration graphs for the determination of molar extinction co-efficient.

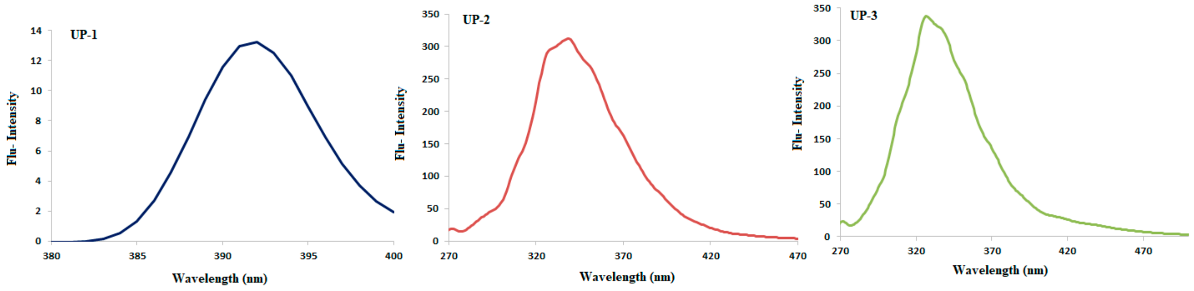

Figure S11. Fluorescence spectrum of compounds UP-1, UP-2 and UP-3.

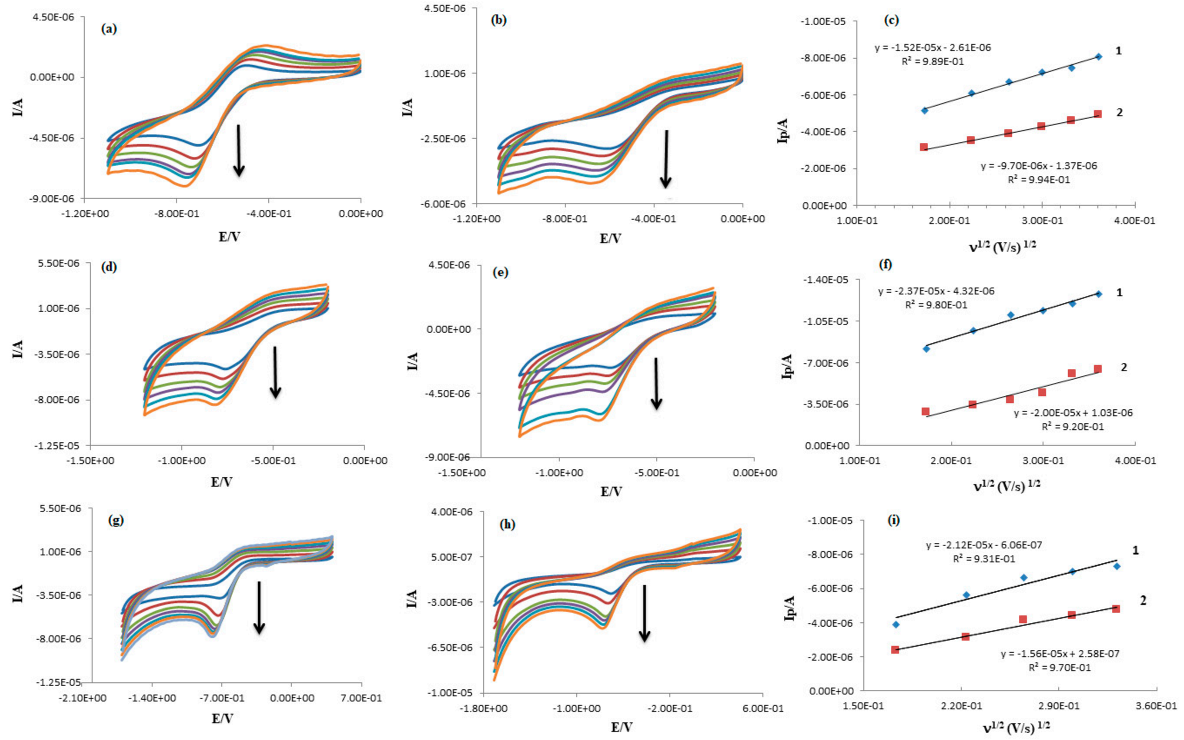

Figure S12. Cyclic voltammetric responses of UP-1, UP-2, and UP-3 without (a,d,g) and with (b,e,h) DNA (60μM) at various scan rates (0.03 - 0.13 V/s), (c,f,i) represents the graphs of Ip vs $\nu^{1/2}$ for the compounds before (1) and after (2) DNA.

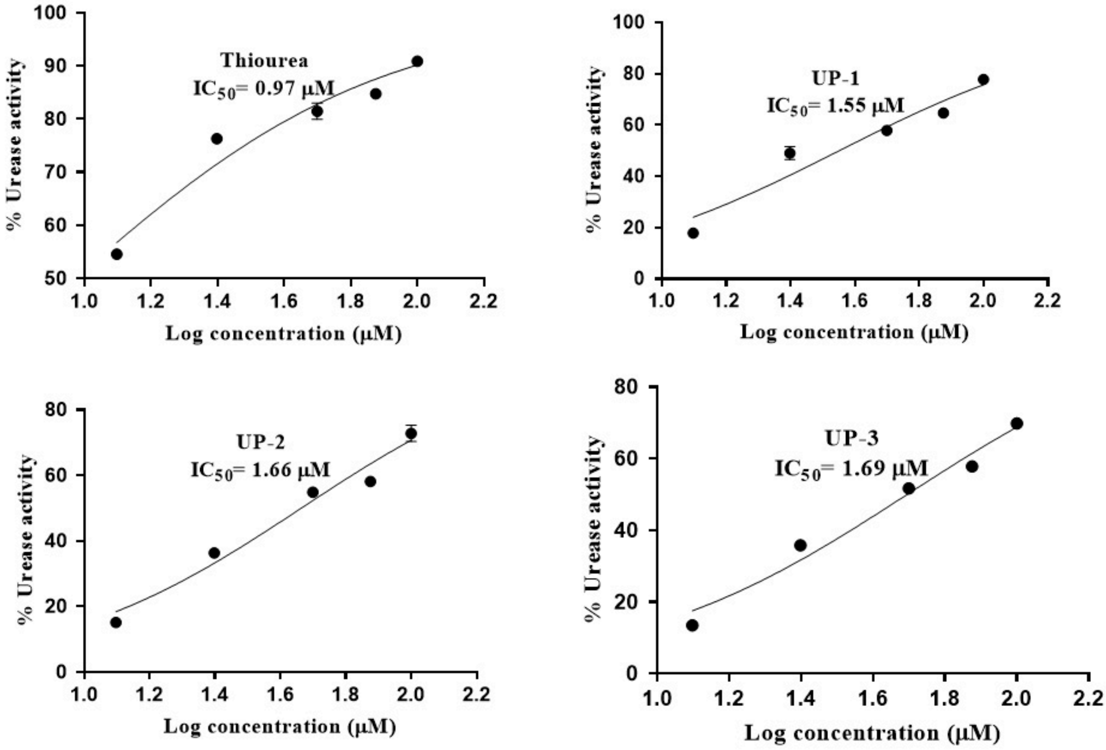

Conc uM	Thiourea			UP-1			UP-2			UP-3		
100	90.91	91.74	90.08	75.65	78.32	79.51	73.1	75.12	70.21	68.96	70.63	69.89
75	85.32	83.98	85.01	63.64	65.29	65.29	58.68	58.84	57.02	57.85	58.68	57.02
50	80.25	83.21	80.99	55.98	58.41	59.22	55.32	54.23	55.09	52.12	51.02	51.89
25	76.86	76.03	76.03	46.28	49.59	51.24	35.54	37.19	36.36	34.71	35.54	37.19
12.5	55.37	54.55	53.72	16.53	18.18	19.01	14.05	15.7	15.7	13.22	14.88	12.4

	Thiourea	UP-1	UP-2	UP-3
LogIC50	0.9708	1.551	1.663	1.694
Std. Error				
LogIC50	0.03712	0.02878	0.01788	0.01615

Figure S13. Graphs for the determination of IC_{50} values of control and investigated compounds.

UP1		
[Compund	ereous MG	rmal HEK-293
40	27.87375	21.821
80	36.50791	44.5535
120	40.18088	52.96915
200	43.76153	59.25645
400	52.99003	69.98483
	Cancereous MG	rmal HEK-293
LogIC50	2.496	2.096
IC50	313.2	124.7
Std. Error		
LogIC50	0.0324	0.04873

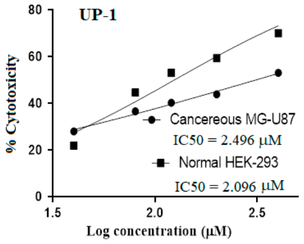

UP2		
[Compund	ereous MG	rmal HEK-293
40μM	10.95212	0
80μM	12.24067	0
120μM	29.44739	0
200μM	39.22111	24.19828
400μM	43.09339	27.19828
LogIC50	2.664	2.856
IC50	461.8	718.5
Std. Error		
LogIC50	0.1298	0.2027

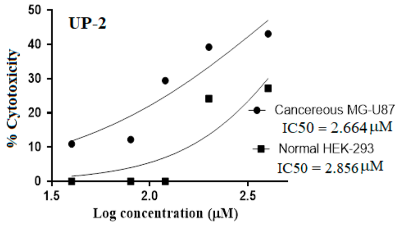

UP3		
[Compund	ereous MG	rmal HEK-293
40μM	17.18346	17.31411
80μM	18.56774	37.49115
120μM	37.04319	44.33485
200μM	41.14064	60.74355
400μM	56.97306	60.97623
LogIC50	2.459	2.22
IC50	287.9	165.9
Std. Error		
LogIC50	0.06558	0.07069

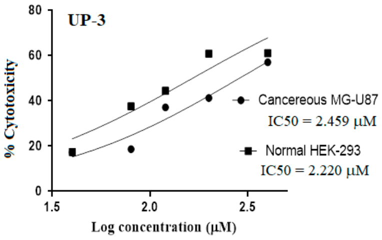

Figure S14. Graphs for the determination of IC_{50} values of investigated compounds against cancerous (MG-U87) and normal (HEK-293) cell lines.
